# Non-Invasive Screening for Early Cognitive Impairment in Elderly Hyperuricaemic Men Using Transcranial Colour-Coded Duplex Sonography

**DOI:** 10.3390/diagnostics15121519

**Published:** 2025-06-15

**Authors:** Zhirong Xu, Jiayi Ye, Han Wang, Jiemin Chen, Kailing Tan, Shilin Li, Shanshan Su

**Affiliations:** 1 Department of Ultrasound, The Second Affiliated Hospital of Fujian Medical University, Quanzhou 362000, China; xzr_fydfe@163.com (Z.X.);; 2 Department of Nuclear Medicine, The Second Affiliated Hospital of Fujian Medical University, Quanzhou 362000, China; yjy_fydfe@163.com

**Keywords:** hyperuricaemia, mild cognitive impairment, transcranial colour-coded duplex, cerebral haemodynamics, third ventricle width

## Abstract

**Objectives:** Hyperuricaemia has been linked to cognitive decline, yet cerebral structural and haemodynamic changes in this population remain poorly defined. We evaluated transcranial colour-coded duplex (TCCD) sonography as a non-invasive screening tool for early mild cognitive impairment (MCI) in elderly hyperuricaemic men. **Methods:** In this cross-sectional study, 195 men aged ≥ 60 years with hyperuricaemia were stratified by the Montreal Cognitive Assessment (MoCA) into HUA + MCI (MoCA < 26, *n* = 46) and HUA (MoCA ≥ 26, *n* = 149) groups. TCCD measured third-ventricle width (TVW) and peak systolic/end-diastolic velocities to calculate resistive (RI) and pulsatility (PI) indices in the middle (MCA) and posterior (PCA) cerebral arteries. Serum uric acid was recorded. Kernel density plots and receiver operating characteristic (ROC) curves assessed diagnostic performance. **Results:** The HUA + MCI group exhibited higher serum uric acid (508.5 ± 36.3 vs. 492.9 ± 44.0 µmol/L; *p* = 0.031), greater TVW (0.55 ± 0.11 vs. 0.51 ± 0.08 cm; *p* = 0.037), and elevated left PCA RI (0.69 ± 0.07 vs. 0.64 ± 0.06) and PI (1.05 ± 0.17 vs. 0.95 ± 0.12; both *p* < 0.001). ROC analysis identified left PCA PI as the most specific marker (AUC = 0.701; specificity 90.6%; sensitivity 45.7%). Kernel density plots confirmed distinct distributions of key parameters. **Conclusions:** TCCD-detected ventricular enlargement and raised PCA pulsatility accurately distinguish MCI among hyperuricaemic men. As a non-invasive, accessible technique with high specificity, TCCD may complement MRI and cognitive testing in early screening of at-risk populations.

## 1. Introduction

Mild cognitive impairment (MCI) is commonly recognized as an early stage of dementia, characterized by a measurable cognitive decline that exceeds normal age-related changes without significantly impairing daily living activities [1]. Given the global ageing trend, the prevalence of MCI is rising, highlighting the urgent need to identify modifiable risk factors and reliable early biomarkers for cognitive impairment [2,3]. Emerging evidence indicates a potential relationship between hyperuricaemia (HUA)—defined as elevated serum uric acid levels—and cognitive dysfunction [4]. Uric acid, the final metabolite in purine metabolism, possesses dual biological roles, serving as an antioxidant extracellularly but potentially exerting pro-oxidative and pro-inflammatory effects under certain pathological conditions [5]. Elevated serum uric acid levels have been associated with cardiovascular risk factors such as hypertension, atherosclerosis, and cerebrovascular disease, all of which negatively influence cognitive function [6,7]. Notably, male patients typically exhibit higher serum uric acid levels and poorer cognitive performance compared to female patients, possibly because oestrogen and progesterone mitigate the detrimental effects of uric acid on cognitive function. Although several studies have linked increased serum uric acid levels to heightened risks of MCI or dementia through mechanisms involving oxidative stress, inflammation, and cerebral small vessel damage [8,9], conflicting findings suggest that the antioxidative properties of uric acid may confer neuroprotection, potentially reducing the risk of cognitive decline [10]. Thus, the current literature presents inconsistent conclusions about the relationship between HUA and cognitive impairment, highlighting the necessity for further research [3]. Clarifying this association is critical not only for a deeper understanding of cognitive impairment pathogenesis but also for guiding preventive and therapeutic strategies in hyperuricaemic populations.

Transcranial colour-coded duplex (TCCD) sonography is a non-invasive neuroimaging technique that enables the real-time assessment of intracranial blood flow dynamics and structural cerebral alterations [11,12]. By transmitting ultrasonic waves through the temporal acoustic window, TCCD measures haemodynamic parameters such as peak systolic velocity (PSV) and end-diastolic velocity (EDV) in major cerebral arteries, including the middle cerebral artery (MCA) and posterior cerebral artery (PCA). It also provides derived indices, particularly the resistive index (RI) and pulsatility index (PI), which reflect cerebrovascular resistance and arterial compliance [13,14]. Elevated PI or RI values suggest increased distal vascular resistance or arterial stiffness, conditions closely associated with cerebral small vessel disease and cognitive impairment [13]. Additionally, TCCD enables the visualization of midline cerebral structures, such as third ventricle width (TVW), a validated ultrasonographic indicator of brain atrophy associated with cognitive decline across various neurodegenerative disorders, including Parkinson’s disease and vascular dementia [12]. Due to its non-invasiveness, affordability, and bedside availability, TCCD is a promising method for the early identification of cerebrovascular and structural brain abnormalities among populations at risk of cognitive decline [15].

Despite the well-documented utility of TCCD in neurological evaluation [11], there is a paucity of research exploring its applicability specifically in hyperuricaemic individuals, particularly concerning cognitive impairment. Previous studies have inadequately addressed whether distinctive cerebral haemodynamic abnormalities or structural brain changes occur in hyperuricaemic patients with MCI. Investigating these parameters using TCCD could illuminate underlying mechanisms linking elevated serum uric acid levels with cognitive deterioration. Therefore, the present study aimed to clarify the relationship between HUA and MCI by evaluating TCCD-derived cerebral haemodynamic parameters and third ventricle width in hyperuricaemic patients with and without MCI. We hypothesized that hyperuricaemic patients with MCI would display increased TVW and abnormal cerebral blood flow indices (e.g., elevated RI or PI values) compared with cognitively normal hyperuricaemic individuals. The overarching objective was to evaluate TCCD’s effectiveness in detecting early cerebral alterations linked to cognitive impairment in hyperuricaemic patients, thereby advancing our understanding of the neurological impact of hyperuricaemia and potentially guiding early clinical intervention.

## 2. Materials and Methods

### 2.1. Study Design and Participants

This cross-sectional observational study was conducted at the Second Affiliated Hospital of Fujian Medical University from January 2021 to March 2025. Ethical approval was granted by the institutional ethics committee, and written informed consent was obtained from all participants or their legal guardians prior to enrolment. A total of 195 male patients aged ≥ 60 years, clinically diagnosed with hyperuricaemia (defined as serum uric acid levels exceeding 420 µmol/L) [5,11], were recruited. All participants had adequate temporal bone acoustic windows, determined by a pre-screening ultrasound to ensure reliable TCCD assessment. Patients were excluded if they had a history of stroke, transient ischaemic attack, dementia, neurodegenerative disorders, severe psychiatric illnesses, substance abuse, acute medical conditions potentially affecting cognitive function, uncontrolled systemic diseases, or poor ultrasound penetration. Cognitive function was evaluated using the Montreal Cognitive Assessment-Beijing version (MoCA-BJ) [16,17]. Participants scoring < 26 points were classified into the HUA + MCI group, indicating mild cognitive impairment, while those scoring ≥ 26 points were categorised into the cognitively normal HUA group. All cognitive assessments using the MoCA-BJ were performed by trained neurologists who were blinded to the TCCD results to avoid assessment bias. Participant inclusion and exclusion were carried out prior to group assignment. No missing data were observed in this study; all patients included had complete datasets.

### 2.2. Equipment and Patient Positioning

All TCCD examinations were performed using a colour Doppler ultrasound system equipped with a 2 MHz phased-array transducer (VISION Avius, Hitachi Medical Systems, Tokyo, Japan). Examinations were conducted by experienced neurosonologists who were blinded to the cognitive status of participants. Subjects were examined in a quiet, temperature-controlled environment and rested supine for at least 10 min before examination to stabilize cerebral haemodynamics. Participants were instructed to avoid caffeine, nicotine, and vasoactive medications for at least 24 h prior to the examination to prevent alterations in cerebral blood flow.

### 2.3. TCCD Examination Procedure

**Figure 1:**
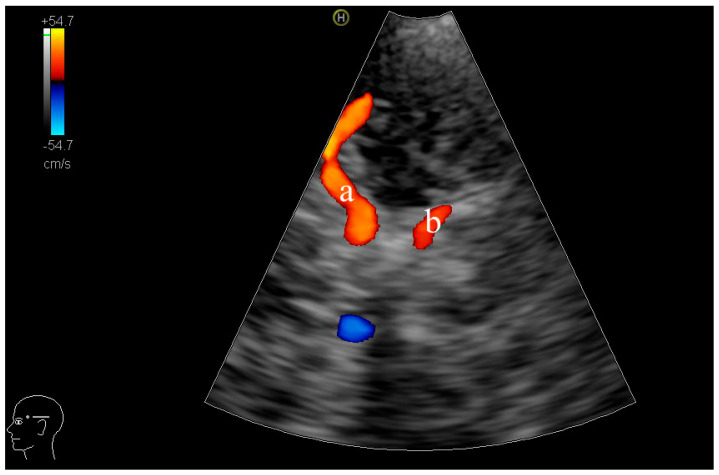
TCCD axial image at the mesencephalic plane. (**a**) indicates the insonation site used for measuring the haemodynamic parameters of the left proximal M1 segment of the MCA, and (**b**) indicates the site used for the left P1 segment of the PCA.

Temporal Acoustic Window: The temporal acoustic window was utilized by positioning the transducer superior to the zygomatic arch and anterior to the tragus. Using an axial insonation plane, bilateral MCA and PCA were identified. The MCA proximal M1 segment was insonated at a depth of 45–55 mm at the mesencephalic or sphenoid ridge plane, clearly identifying midbrain landmarks [18]. The PCA P1 segment was examined at the mesencephalic plane at approximately 60–70 mm [19]. Vessels were identified based on anatomical landmarks and colour-coded Doppler flow signals, with MCA M1 and PCA P1 segments typically demonstrating flow directed toward the transducer. The insonation sites for the M1 and P1 segments are shown in Figure 1a and Figure 1b, respectively.

TVW: TVW was measured using B-mode ultrasound imaging at the axial midline thalamic level. TVW was defined as the maximal perpendicular distance between the inner echogenic borders of the third ventricle. Each participant’s TVW measurement represented the mean value obtained from three consecutive, stable ultrasound measurements.

Haemodynamic Parameters: Spectral Doppler waveforms were recorded bilaterally from the MCA-M1 and PCA-P1 segments. PSV and EDV were measured from clear and stable spectral Doppler waveforms obtained during at least five consecutive cardiac cycles. Additionally, RI and PI were calculated according to standard formulas:

RI = (PSV − EDV)/PSV  PI = (PSV − EDV)/Mean Velocity

The mean velocity was automatically computed by the ultrasound system based on the recorded Doppler waveforms.

Ultrasound Settings Standardisation: To ensure consistency and comparability across examinations, the Doppler insonation angle was consistently maintained ≤30°, or angle correction was applied if available. The sample volume was uniformly set at 4 mm for both MCA and PCA measurements. Colour gain was adjusted to a point just below the threshold at which noise artifacts appeared, and pulse repetition frequency (PRF) was typically set between 5 and 7 kHz to avoid aliasing and obtain clear spectral waveforms. Depth, overall gain, and focal zone settings were standardized for all participants to minimize variability between examinations [20].

### 2.4. Measurement Reproducibility

To confirm measurement reproducibility, 20 randomly selected participants underwent reliability assessments. Intra-observer reliability was evaluated through repeated measurements performed by the same examiner one week apart, while inter-observer reliability was assessed by two independent examiners conducting examinations within one hour on the same day. Intraclass correlation coefficients (ICCs) were calculated, with values > 0.75 indicative of excellent reliability, thereby ensuring robust and reproducible measurements.

### 2.5. Statistical Analysis

Data analysis was performed using SPSS 26.0 (IBM Corp., Armonk, NY, USA) and R version 4.3.3 (R Foundation for Statistical Computing, Vienna, Austria). Continuous variables were assessed for normality using the Shapiro–Wilk test. Normally distributed data were expressed as means ± standard deviations (SD), and non-normally distributed data were reported as medians and interquartile ranges (IQR). Group comparisons were conducted using Student’s *t*-test or Mann–Whitney U test, as appropriate. Categorical variables were compared using the chi-square test or Fisher’s exact test.

Receiver operating characteristic (ROC) curves were generated to evaluate the diagnostic accuracy of serum uric acid, third ventricle width, and TCCD-derived indices. The area under the curve (AUC), sensitivity, and specificity were calculated. To visually compare the distributions of key variables between the HUA and HUA + MCI groups, kernel density distribution plots were constructed using the ggplot2 and ggpubr packages in R. These plots illustrated differences in the distribution and central tendency of clinical and ultrasound parameters across groups. All statistical tests were two-tailed, and a *p*-value < 0.05 was considered statistically significant.

## 3. Results

### 3.1. Clinical Characteristics and Serum Uric Acid Levels

Baseline clinical characteristics and vascular risk factors of participants are presented in Table 1. Age and common vascular risk factors were comparable between the two groups. Notably, patients in the HUA + MCI group exhibited significantly higher mean serum uric acid levels compared to cognitively normal hyperuricaemic participants (508.48 ± 36.25 vs. 492.93 ± 44.01 μmol/L, *p* = 0.031). Despite all subjects having elevated uric acid, this observed difference underscores a potential relationship between greater serum uric acid elevations and cognitive impairment. Other comorbid conditions, including hypertension, diabetes, hyperlipidaemia, and body mass index, were similar across groups (all *p* > 0.05), confirming adequate baseline comparability aside from uric acid concentrations.

### 3.2. TCCD Findings: Structural and Haemodynamic Differences

TCCD identified distinct structural and haemodynamic alterations between the two groups (Table 2). Structurally, patients in the HUA + MCI group showed significant enlargement of the third ventricle compared to the cognitively normal HUA group (0.55 ± 0.11 cm vs. 0.51 ± 0.08 cm, *p* = 0.037). Representative TCCD images demonstrated visibly increased third ventricle widths in patients with cognitive impairment. Haemodynamic assessments further revealed marked differences predominantly in PCA indices. Specifically, left PCA RI and PI were significantly elevated in the HUA + MCI group compared to cognitively normal patients (RI: 0.69 ± 0.07 vs. 0.64 ± 0.06; PI: 1.05 ± 0.17 vs. 0.95 ± 0.12, both *p* = 0.0001). These findings suggest increased intracranial vascular resistance primarily affecting the posterior cerebral circulation in MCI patients. In contrast, haemodynamic parameters of the bilateral MCA—including peak systolic velocity, end-diastolic velocity, RI, and PI—showed no significant differences between groups (all *p* > 0.1; Table 2). Thus, cerebral haemodynamic changes associated with MCI appear to preferentially involve posterior rather than anterior circulation.

**Figure 2:**
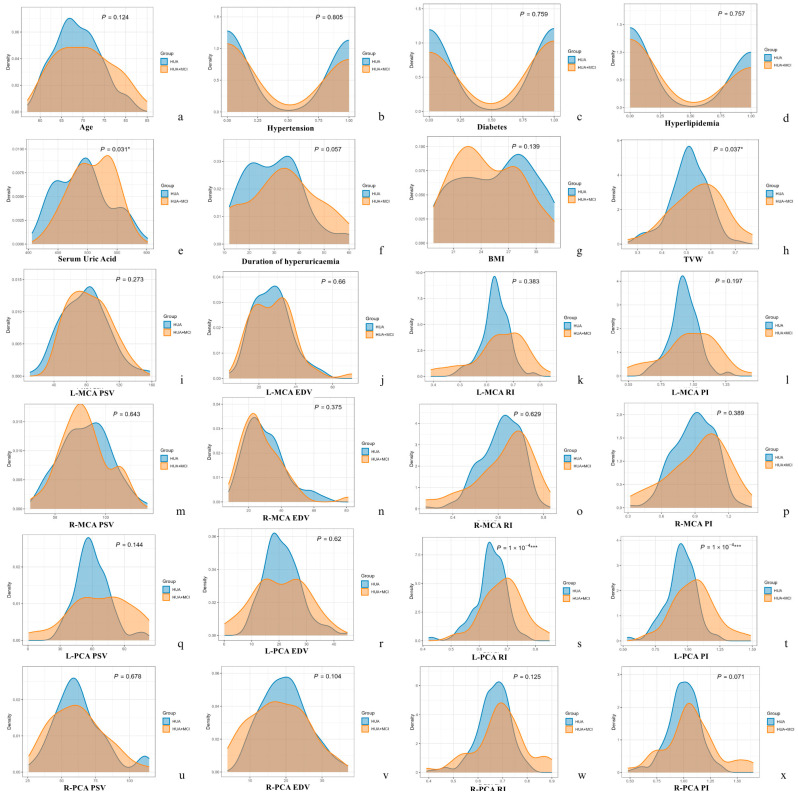
Kernel density distribution plots comparing clinical and transcranial colour-coded duplex (TCCD) parameters between hyperuricaemia patients with and without mild cognitive impairment (MCI). Each subplot (**a**–**x**) represents a specific variable: (**a**) Age, (**b**) Hypertension, (**c**) Diabetes, (**d**) Hyperlipidaemia, (**e**) Serum uric acid, (**f**) Duration of hyperuricaemia, (**g**) Body mass index (BMI), (**h**) Third ventricle width (TVW), (**i**–**l**) Left middle cerebral artery (MCA): (**i**) PSV, (**j**) EDV, (**k**) RI, (**l**) PI; (**m**–**p**) Right MCA: (**m**) PSV, (**n**) EDV, (**o**) RI, (**p**) PI; (**q**–**t**) Left posterior cerebral artery (PCA): (**q**) PSV, (**r**) EDV, (**s**) RI, (**t**) PI; (**u**–**x**) Right PCA: (**u**) PSV, (**v**) EDV, (**w**) RI, (**x**) PI. The blue curves represent the cognitively normal HUA group, while the orange curves represent the HUA + MCI group. Statistically significant differences were observed in serum uric acid (**e**), third ventricle width (**h**), and left PCA resistive index (**s**) and pulsatility index (**t**), all with *p*-values < 0.05. Other parameters, including MCA measurements and systemic comorbidities, did not show significant intergroup differences (all *p* > 0.05). These distributions highlight the specific TCCD and clinical variables that distinguish cognitive status in hyperuricaemic individuals. Note: * *p* < 0.05; *** *p* < 0.001.

Kernel density distribution plots further illustrated the distribution differences of clinical and ultrasonographic parameters between the HUA and HUA + MCI groups (Figure 2). Notably, significant differences were visually apparent for serum uric acid (*p* = 0.031), third ventricle width (*p* = 0.037), and left PCA indices including RI and PI (both *p* < 0.0001). Other parameters displayed overlapping distributions with no statistically significant differences between groups, reinforcing that key distinguishing features between cognitively impaired and normal hyperuricaemic patients included elevated serum uric acid, enlarged third ventricle width, and increased vascular resistance within the left PCA.

### 3.3. Diagnostic Accuracy of TCCD Parameters for MCI

**Figure 3:**
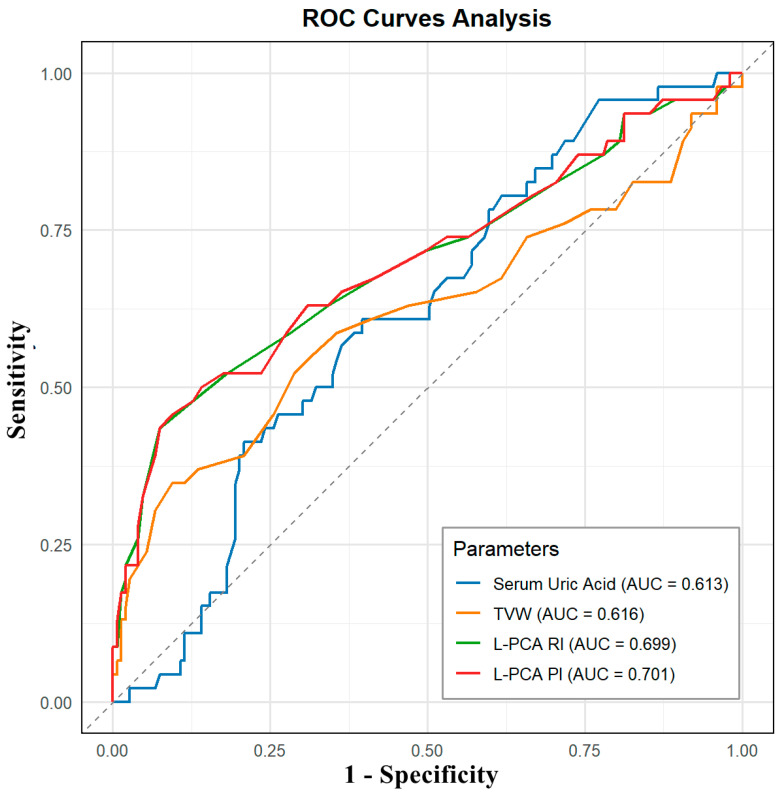
ROC curves for identifying MCI in hyperuricaemic patients using serum uric acid, TVW, and TCCD-derived indices from the left PCA. Serum uric acid showed modest discrimination (AUC = 0.613), while TVW had a similar performance (AUC = 0.616). Left PCA RI and PI demonstrated higher diagnostic accuracy, with AUCs of 0.699 and 0.701, respectively. Among all parameters, left PCA PI and RI showed the highest specificity. The diagonal line indicates the line of no discrimination.

Receiver operating characteristic (ROC) analyses evaluated the discriminatory performance of serum uric acid and significant TCCD-derived parameters in identifying MCI among hyperuricaemic patients (Table 3, Figure 3). Serum uric acid alone displayed modest predictive accuracy (AUC = 0.613), achieving a sensitivity of 60.87% and specificity of 60.40% at an optimal threshold of 500.5 μmol/L. The third ventricle width similarly exhibited modest diagnostic potential (AUC = 0.616); however, its optimal cutoff (0.605 cm) was highly specific (90.6%) but had limited sensitivity (34.8%) for MCI detection. Importantly, TCCD-derived left PCA indices demonstrated superior diagnostic capabilities. Left PCA RI yielded an AUC of 0.699, with a sensitivity of 43.48% and high specificity of 92.62% at a cutoff value of 0.705. Likewise, left PCA PI presented the highest discriminatory performance (AUC = 0.701), providing sensitivity of 45.65% and specificity of 90.60% at an optimal cutoff of 1.075. As illustrated in Figure 3, ROC curves for PCA PI and RI parameters were notably superior compared to serum uric acid and third ventricle width. Although the sensitivity of TCCD parameters was moderate, their high specificity suggests that elevated PCA RI and PI values are robust markers indicating cognitive impairment in hyperuricaemic individuals. Collectively, these findings indicate that hyperuricaemic patients with MCI are characterised by elevated serum uric acid, structural enlargement of the third ventricle, and distinctive cerebral haemodynamic changes detectable via TCCD—particularly increased PCA RI and PI—with the latter offering the highest diagnostic specificity for early cognitive impairment detection.

## 4. Discussion

This study identified distinct cerebral sonographic differences between elderly hyperuricaemic men with and without MCI. Compared to cognitively normal counterparts, those with MCI exhibited significantly greater TVW and elevated RI and PI in the left PCA, suggesting both structural brain atrophy and altered cerebral haemodynamics [21,22]. Additionally, serum uric acid levels were significantly higher in the MCI group, despite all participants being clinically hyperuricaemic. ROC analyses further demonstrated that left PCA PI had the greatest discriminatory ability among all evaluated parameters, underscoring the potential of cerebral haemodynamic markers in identifying cognitive impairment within this population [23].

These findings contribute to the ongoing debate concerning the role of uric acid in cognitive health. While some studies have highlighted its neuroprotective potential due to antioxidant properties, others have reported associations with inflammation, oxidative stress, and cerebrovascular damage [24,25]. Notably, moderate elevations in uric acid within the normal range have been linked to reduced cognitive decline, yet persistent hyperuricaemia appears to accelerate neurovascular degeneration. Our results align with the latter, indicating that even within a hyperuricaemic cohort, higher serum uric acid levels are associated with MCI. This supports the hypothesis that chronic uric acid elevation contributes to vascular dysfunction rather than offering cognitive protection [26].

Beyond biochemical markers, our study offers new insight into cerebrovascular changes detectable by TCCD. The observed enlargement of the third ventricle in the MCI group reflects brain atrophy, consistent with previous findings in neurodegenerative and vascular cognitive disorders [27,28]. While ventricular enlargement has been extensively documented via MRI, our data confirm that TCCD offers a reliable and accessible alternative for detecting early structural changes, particularly in at-risk populations such as hyperuricaemic patients.

In addition to structural indicators, we identified significant haemodynamic abnormalities, specifically increasing RI and PI in the left PCA. These indices are typically interpreted as markers of increased downstream vascular resistance or impaired autoregulation, both features of cerebral small vessel disease [29]. Our findings align with previous studies in hypertensive and Alzheimer’s populations, which have reported similar associations between elevated pulsatility and cognitive dysfunction [30]. The lateralised pattern of the changes, primarily involving the left PCA, is particularly noteworthy and may point to hemispheric asymmetries in vulnerability to metabolic or vascular insults—an area deserving further exploration [31].

From a clinical perspective, our results highlight the utility of TCCD as a non-invasive adjunctive tool for the early detection of cognitive changes in patients with metabolic risk factors. Unlike MRI, TCCD can be performed at the bedside and repeated as needed, making it highly suitable for longitudinal monitoring. Parameters such as TVW and PCA PI may serve as early warning signs, enabling timely intervention. For clinicians managing hyperuricaemic patients, incorporating routine TCCD assessments could facilitate identification of those at increased risk for cognitive impairment, prompting earlier lifestyle or pharmacological interventions.

Several limitations should be acknowledged. The cross-sectional nature of the study restricts causal inference. Additionally, the sample consisted exclusively of elderly males from a single centre, limiting the generalisability of findings. The requirement for adequate acoustic windows may have introduced selection bias by excluding patients with suboptimal imaging conditions. Future longitudinal studies with larger, more diverse populations and integrated multimodal imaging approaches are warranted to validate our findings, establish causality, and refine the predictive value of TCCD parameters in cognitive assessment.

In addition, the study did not include a healthy non-hyperuricaemic control group, which limits the ability to determine how TCCD parameters in hyperuricaemic patients compare to those with normal uric acid levels. Future studies incorporating such a control group could provide more robust reference values and enhance the clinical interpretability of TCCD findings. Additionally, we did not assess how the autonomic nervous system may affect cerebral blood flow in this study. This regulatory mechanism could be relevant to cognitive function and brain perfusion, and may be considered in future research using neurovascular imaging or physiological monitoring methods such as heart rate variability.

## 5. Conclusions

This study demonstrates that hyperuricaemic patients with MCI show clear signs of structural brain atrophy and increased cerebrovascular resistance as measured by TCCD, along with higher serum uric acid levels. These results support the presence of a cerebrovascular mechanism underlying cognitive impairment in this population. Routine cerebral ultrasonography may serve as a valuable adjunct to existing diagnostic approaches into the clinical assessment of hyperuricaemic patients, and future research should aim to explore preventive strategies targeting cerebral perfusion and metabolic control.

## Figures and Tables

**Table 1:** Baseline clinical characteristics of hyperuricaemic patients with and without MCI.

Parameters	HUA Group (*n* = 149)	HUA + MCI Group (*n* = 46)	*p* Value
Age (year, X ± SD)	68.70 ± 5.29	70.37 ± 6.62	0.124
Serum uric acid (μmol/L, X ± SD)	492.93 ± 44.01	508.48 ± 36.25	0.031 *
Hypertension Duration (month, X ± SD)	30.04 ± 11.19	33.87 ± 13.77	0.057
Hypertension (*n*, %)	70, 47.0	20, 43.5	0.805
Diabetes (*n*, %)	75, 50.3	25, 54.3	0.759
Hyperlipidaemia (*n*, %)	61, 40.9	17, 37.0	0.757
BMI (X, X ± SD)	25.7 ± 3.9	24.7 ± 3.5	0.139

HUA, hyperuricemia MCI, mild cognitive impairment; BMI, body mass index. * *p* < 0.05.

**Table 2:** Comparison of TCCD-Derived Cerebral Haemodynamic and Structural Parameters Between HUA and HUA + MCI Groups.

Parameters		HUA Group	HUA + MCI Group	*p* Value
TVW (cm)		0.51 ± 0.08	0.55 ± 0.11	0.037 *
Left MCA	PSV (cm/s)	78.06 ± 27.70	83.08 ± 25.05	0.273
	EDV (cm/s)	27.03 ± 10.16	27.81 ± 11.79	0.660
	RI	0.63 ± 0.05	0.65 ± 0.10	0.383
	PI	0.93 ± 0.11	0.97 ± 0.21	0.197
Right MCA	PSV (cm/s)	80.50 ± 23.88	78.65 ± 23.01	0.643
	EDV (cm/s)	29.94 ± 12.17	28.08 ± 13.10	0.375
	RI	0.61 ± 0.09	0.62 ± 0.13	0.629
	PI	0.89 ± 0.18	0.93 ± 0.26	0.389
Left PCA	PSV (cm/s)	60.45 ± 15.90	66.99 ± 28.59	0.144
	EDV (cm/s)	21.18 ± 6.61	20.42 ± 9.70	0.620
	RI	0.64 ± 0.06	0.69 ± 0.07	0.0001 ***
	PI	0.95 ± 0.12	1.05 ± 0.17	0.0001 ***
Right PCA	PSV (cm/s)	63.19 ± 18.66	61.85 ± 19.75	0.678
	EDV (cm/s)	19.73 ± 6.41	17.87 ± 7.74	0.104
	RI	0.66 ± 0.07	0.69 ± 0.07	0.125
	PI	1.00 ± 0.15	1.07 ± 0.25	0.071

MCA, middle cerebral artery; PCA, posterior cerebral artery; PSV, peak systolic velocity; EDV, end diastolic velocity; RI, resistance index; PI, pulsatility index. * *p* < 0.05 and *** *p* < 0.001 compared with HUA group.

**Table 3:** Diagnostic Performance of Serum Uric Acid and TCCD Parameters for Predicting MCI in Hyperuricaemic Patients.

Parameter	AUC	Sensitivity	Specificity	Threshold
Serum Uric Acid	0.613	60.87%	60.40%	500.500
TVW	0.616	34.78%	90.60%	0.605
L-PCA RI	0.699	43.48%	92.62%	0.705
L-PCA PI	0.701	45.65%	90.60%	1.075

AUC, area under the curve.

## Data Availability

The datasets used and analysed during this study are available from the corresponding author on reasonable request.

## Abbreviations

The following abbreviations are used in this manuscript:

MCI	mild cognitive impairment
HUA	hyperuricaemia
TCCD	transcranial colour-coded duplex
PSV	peak systolic velocity
EDV	end-diastolic velocity
MCA	middle cerebral artery
PCA	posterior cerebral artery
RI	resistive index
PI	pulsatility index
TVW	third ventricle width
MoCA	Montreal Cognitive Assessment
ICC	intraclass correlation coefficient
SPSS	Statistical Package for the Social Sciences
ROC	receiver operating characteristic
AUC	area under the ROC curve
BMI	body mass index
PRF	pulse repetition frequency
